# Nutriomic Technologies for Characterizing, Diagnosing, Clustering and Managing Chronic Liver Diseases: Precision Nutrition Implications

**DOI:** 10.3390/ijms27188106

**Published:** 2026-09-11

**Authors:** Nuria Perez-Diaz-del-Campo, Miguel Lopez-Moreno, Begoña de Cuevillas, Diego Martinez-Urbistondo, J. Alfredo Martínez, Omar Ramos-Lopez

**Affiliations:** 1 Diet, Planetary Health and Performance, Faculty of Health Sciences, Universidad Francisco de Vitoria, 28223 Pozuelo, Spain; nuria.perez@ufv.es (N.P.-D.-d.-C.); miguel.lopez@ufv.es (M.L.-M.); 2 Institute of Health and Sport Sciences, Faculty of Health Sciences, Universidad Francisco de Vitoria, 28223 Madrid, Spain; 3 Área de Medicina Vascular, Departamento de Endocrinología, Clínica Universidad de Navarra, 28027 Madrid, Spain; begona.decuevillas@nutricion.imdea.org; 4 Internal Medicine Department, Clínica Universidad de Navarra, 28027 Madrid, Spain; dmurbistondo@gmail.com; 5 Centro de Investigacion Biomedica en Red Area de Fisiologia de la Obesidad y la Nutricion [CIBEROBN], 28029 Madrid, Spain; jalfredo.martinez@nutricion.imdea.org; 6 Precision Nutrition and Cardiometabolic Health Program, IMDEA Nutrition [Madrid Institute for Advanced Studies], 28049 Madrid, Spain; 7 Department of Medicine and Endocrinology, University of Valladolid, 47002 Valladolid, Spain; 8 Medicine and Psychology School, Autonomous University of Baja California, Tijuana 22390, Mexico

**Keywords:** precision nutrition, nutritional omics, chronic liver disease, MASLD, MASH, metabolic liver disease

## Abstract

Chronic liver diseases are frequently accompanied by metabolic dysfunction, making precision nutrition relevant for prevention, diagnosis, risk stratification, and management. This review summarizes nutritional omics evidence in hepatology, emphasizing metabolic dysfunction-associated steatotic liver disease as a prevalent model. Nutrigenetics provides information on inherited susceptibility by identifying genetic variation affecting hepatic lipid handling, triglyceride export, phospholipid remodeling, and glucose-driven lipogenesis. Nutrigenomics characterizes transcriptional programs involved in hepatic lipogenesis, inflammation, oxidative stress, and fibrogenesis. Nutriepigenetics captures exposure memory through DNA methylation, histone regulation, and small-RNA signaling, including miR-122, miR-21, miR-34a, and miR-192; diet may modulate this layer through one-carbon metabolism, methyl-donor availability, oxidative stress, acetyl-CoA and NAD+-dependent pathways, and lipid peroxidation. Nutrimetagenomics highlight taxa such as Ruminococcus, Faecalibacterium, Veillonella, Bacteroides, Escherichia, and Klebsiella, but translation requires functional characterization beyond stool taxa. Nutrimetabolomics and lipidomics, including OWL-liver platforms, track dietary adherence, lipid species, bile acids, amino acids, lipoproteins, and biological non-response. Future progress will require AI-supported, phenotype-first integrated models tested in multiethnic longitudinal studies with standardized dietary assessment, biospecimen collection, meaningful liver endpoints, realistic workflows, and adaptive lifestyle-care strategies.

## 1. Introduction

The modern burden of liver disease is both economically costly and physiopathologically dynamic concerning processes and outcomes [1,2]. A recent GBD-based analysis estimated that major chronic liver diseases together affected about 1.7 billion people worldwide in 2021 [3], while a companion analysis reported 58.4 million incident cases, 1.43 million deaths, and 46.4 million DALYs from cirrhosis and other chronic liver diseases in the same year [4]. The epidemiological focus is shifting, with the incidence and prevalence of hepatitis B and hepatitis C declining in many regions, while MASLD, cirrhosis, and other chronic liver diseases have increased [3,4]. MASLD-related cirrhosis was the only major cause of cirrhosis whose age-standardized incidence increased in recent analyses, partly explaining why metabolic liver disease now dominates discussions on precision nutrition within the field of hepatology [4,5]. The prevalence of MASLD is currently estimated at approximately 38% among adults and is projected to exceed 55% by 2040, suggesting that future strategies for liver disease control will increasingly rely on effective metabolic and nutritional management rather than exclusively on antiviral or abstinence-based approaches [6,7,8]. Precision nutrition provides the conceptual framework for this personalization, seeking to integrate genetic, metabolic, metagenomic, epigenetic, physiopathological, behavioral, sociocultural, medical-history, lifestyle, environmental, and cultural information and factors related to nutriomics to explain inter-individual variability in dietary responses and to tailor nutritional strategies, wellbeing monitoring, and health actions to individuals or population subgroups [9,10]. In this context, the 4P framework refers to predictive, preventive, personalized, and participatory nutrition, emphasizing the use of molecular and clinical information to anticipate risk, prevent progression, tailor interventions, and actively involve patients in long-term lifestyle management [11]. These settings are particularly relevant in liver disease because hepatic steatosis, inflammation, and fibrosis are not medical equivalent end points [12].

The economic consequences are equally important concerning liver disease treatments [13]. A recent global framework placed annual direct medical costs of MASLD at enormous levels in the United States and Europe, and a 2025 population-based study from a Spanish Mediterranean region confirmed that MASLD is associated with substantial healthcare use and measurable economic burden in routine care [14]. Therefore, the case for precision nutrition is not only biological but also health system-based: if inexpensive phenotyping can identify those patients that need more intensive dietary support, or which biomarkers can replace repeated expensive imaging or biopsy in parts of follow-up, the clinical and economic payoff could be considerable [15]. Therefore, precision nutrition has relevance beyond the concurrent biological rationale, extending to health system efficiency: by using low-cost phenotyping to identify patients who require more intensive dietary support, predict diagnostic or progression risk, and prioritize non-invasive biomarkers [16] over repeated imaging or biopsy, which could generate substantial clinical and economic benefits [15,17,18].

Personalization of nutrition in liver disease is warranted because hepatic steatosis, inflammation, and fibrosis are not nutritionally equivalent end points among subjects but present interindividual differences [12]. Some patients appear to have predominantly genetically mediated hepatic lipid trapping, whereas others show dominant adipose-tissue insulin resistance, microbiome-mediated endotoxemia, or profound alterations in amino acid and lipid metabolism [19]. This heterogeneity suggests that the probabilistic benefit of dietary interventions is likely to vary according to the molecular context [20,21]. Although MASLD/MASH currently represents the most extensively studied model for nutritional omics and precision nutrition, similar principles may also be relevant to other chronic liver diseases, including alcohol-related liver disease, viral hepatitis, cholestatic disorders, and cirrhosis, in which the nutritional status, metabolic alterations, inflammatory pathways, and host–microbiome interactions may also influence disease progression and treatment response. Precision nutrition for these conditions remains conceptually important but empirically less developed [22,23]. Therefore, the aim of this review is to provide a comprehensive overview of the current evidence supporting precision nutrition in liver disease, with a particular focus not only on MASLD/MASH as the most extensively studied model but also on the potential benefits of omics approaches to understand and precisely manage liver disease as coordinated with deep phenotypes.

**Figure 1:**
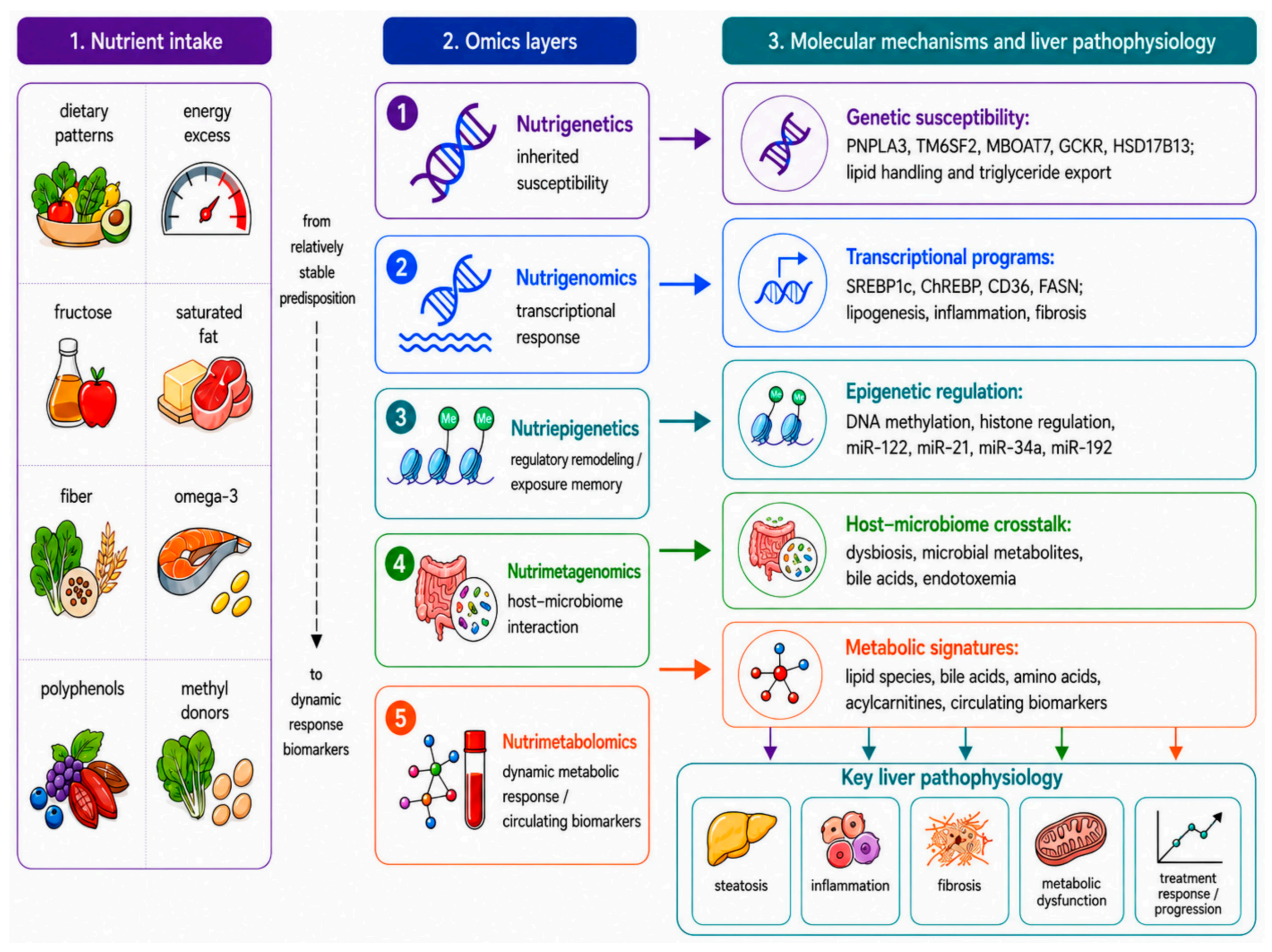
Conceptual framework of nutritional omics in chronic liver disease. The figure summarizes how nutrient intake is linked to complementary omics layers and how these layers relate to molecular mechanisms involved in liver pathophysiology, including steatosis, inflammation, fibrosis, metabolic dysfunction, and treatment response/progression.

To facilitate an integrated understanding of the field, Figure 1 presents a conceptual framework linking nutrient intake, omics layers, and the molecular mechanisms involved in liver pathophysiology in chronic liver disease, with particular emphasis on MASLD/MASH.

## 2. Nutritional Omics in Chronic Liver Disease

The term nutritional omics refers to a cluster of complementary disciplines that seek to explain that dietary exposures do not act uniformly across individuals, tissues, and disease stages, and because some patients improve profoundly with lifestyle intervention whereas others show persistent steatosis, inflammation, or fibrosis despite apparently similar counseling [24,25]. In chronic liver disease, and especially in MASLD/MASH, this framework is particularly compelling because the liver is both a metabolic hub and a nutrient-sensing organ that integrates signals from adipose tissue, skeletal muscle, the intestine, circulating metabolites, and immune pathways [26]. The practical consequence is that “diet” can no longer be treated as a single exposure, because the calorie load, nutrient composition, meal patterning, gut microbial processing, inherited susceptibility, and biological response all modify the liver response to nutritional inputs [22]. Therefore, nutritional omics should be presented not as a collection of technologies, but as a hierarchy of tools that can refine who is at risk, which mechanism is dominant, which biomarker is most sensitive, and whether a lifestyle intervention is producing an adequate biological response [23].

The main challenge is that the omics fields differ markedly in maturity, reproducibility, and proximity to routine practice [27] and need to be integrated. Some signals, such as susceptibility associated with patatin-like phospholipase domain-containing protein 3 (*PNPLA3*), a key genetic determinant of hepatic fat accumulation and MASLD progression, or metabolite-based dietary signatures, have comparatively clear translational narratives [28,29]. Others, such as liver epitranscriptomic remodeling or stool taxonomic response patterns, are mechanistically important but still difficult to translate into stable, low-burden clinical workflows [30,31]. It is therefore more appropriate to view this field as a process comprising increasingly relevant steps: diagnosis risk stratification, endotype definition and response monitoring, as graphically reported [32].

### 2.1. Nutrigenetics

Nutrigenetics examines the role of inherited variation as a modifier of the effect of nutrients, food patterns, or lifestyle interventions on disease susceptibility and therapeutic response [27]. In MASLD, this is the omics layer with the clearest biological basis, because the leading risk loci converge on precisely the hepatic pathways that make nutrition clinically relevant, including lipid-droplet remodeling, triglyceride export, phospholipid remodeling, and glucose-driven de novo lipogenesis [33]. The most frequently discussed variants remain *PNPLA3*, *TM6SF2*, *MBOAT7*, *GCKR* and *HSD17B13* (Table 1). Together, they illustrate that inherited susceptibility or protection may arise from impaired mobilization of stored fat, defective VLDL secretion, altered membrane phospholipid composition, enhanced substrate delivery into lipogenic pathways, or modulation of hepatocellular injury and disease progression [27,34]. From a precision-nutrition perspective, this issue matters because individuals carrying these variants may not experience the same hepatocellular consequences when exposed to excess calories, fructose-rich diets, saturated fats, or unfavorable dietary patterns [33].

A major attraction of nutrigenetics is methodological stability [35]. Genotype is fixed, can be measured once, and is not distorted by short-term dietary recall bias, acute illness, or transient changes in aminotransferases [27,36]. This framework means nutrigenetics are well suited to baseline enrichment, meaning the identification of patients whose metabolic livers are more vulnerable and may therefore merit earlier counseling, closer imaging follow-up, or lower thresholds for additional metabolic intervention [28]. This translational role has been supported by precision-nutrition studies, where genetic information has been combined with phenotypic and environmental variables to predict differential responses to energy-restricted diets [37]. An integrative prototype using genetic, phenotypic, and environmental information for the personalized prescription of energy-restricted diets showed that genetic data are most informative when interpreted together with clinical and lifestyle context rather than as isolated variants [37]. In the liver-specific setting, data from the Fatty Liver in Obesity [FLiO] study showed that genetic risk scores and selected variants may contribute to the stratification of patients with NAFLD undergoing energy-restricted dietary treatment. In particular, genetic risk scores based on the fatty liver index, MRI, and lipidomic traits were proposed for nutrigenetic management of NAFLD, while *SH2B1* rs7359397 was associated with a differential response to a six-month energy-restricted intervention [38,39]. These findings reinforce the idea that nutrigenetics in MASLD should be used to refine diagnostic and risk stratification, as well as treatment intensity, especially when combined with insulin resistance markers and other clinical variables, rather than prescribing diets from the genotype alone [40]. However, concurrent apparent simplicity is deceptive, because the same genotype can behave differently across ancestries, adiposity states, background diets, and disease stages [41]. For that reason, the central translational question is no longer whether genes matter in MASLD, because that is beyond doubt, but whether gene–diet interactions are large, consistent, and reproducible to justify differential dietary advice at the bedside [42].

In a population-based cohort, poor diet quality amplified the unfavorable effect of genetic risk on hepatic steatosis and fibroinflammatory liver phenotypes, implying that diet quality matters even more in genetically susceptible individuals rather than less [28]. This outcome is clinically important because it shifts the focus away from a fatalistic view around “bad genes” and towards a model of mutual interactions where an unhealthy diet and hereditary risk reinforce one another, rather than being mutually exclusive [28]. In a Korean cohort of 33,133 adults, Mediterranean-diet adherence interacted with genetic susceptibility in relation to MASLD risk, strengthening the argument that diet quality is not biologically equivalent across all genomic backgrounds [41]. Similarly, analysis of the Korean Genome and Epidemiology Study suggested nutrient–*PNPLA3* interactions, although the culturally specific dietary signals emerging from that work should be interpreted as hypothesis-generating rather than prescriptive because residual pattern confounding remains plausible [33].

The interventional literature provides the most important corrective to overstatement [42]. A randomized controlled trial published in 2025 found that Mediterranean and low-fat hypocaloric diets were similarly effective for short-term MASLD resolution regardless of *PNPLA3* genotype [42]. When two dietary patterns share the key therapeutic feature of caloric restriction with broadly healthy food quality, a single common variant may not be powerful enough to dictate which one should be prescribed [27]. In this sense, nutrigenetics currently appears more useful for identifying susceptibility and intensity of follow-up than for selecting between two already evidence-based hypocaloric dietary templates [28,42].

Despite the growing interest in nutrigenetics as a tool for precision hepatology, clinical interpretations require caution. Most gene–diet studies are still observational and may be affected by residual confounding related to total energy intake, obesity, socioeconomic factors, and the inherent limitations of dietary assessment methods [41]. In addition, many reported associations appear to be ancestry-dependent, limiting the assumption that findings can be directly extrapolated across populations [33]. The predictive contribution of isolated genetic variants beyond established clinical factors such as obesity, diabetes, and routine fibrosis markers also remains modest [27,36]. Moreover, although evidence supporting variant-specific dietary or therapeutic interventions in MASLD remains still limited, nutrigenetic intervention studies in obesity-related metabolic risk have shown that genome-informed dietary strategies may improve anthropometric, lipid, and inflammatory biomarkers, supporting their potential relevance for metabolic liver disease contexts [42,43,44]. For these reasons, the clinically meaningful approach is likely to rely on polygenic or pathway-level risk scores rather than on the interpretation of single SNPs in isolation [28]. At present, the most reasonable application of nutrigenetics is therefore to support diagnosis-oriented risk stratification, helping to identify patients with a higher probability of clinically relevant steatosis, advanced fibrosis, or progressive MASLD who may benefit from earlier imaging, non-invasive fibrosis testing, more intensive lifestyle counseling, and closer metabolic follow-up. However, current evidence does not support translating each genotype into a distinct dietary prescription; rather, genetic information should be interpreted as an adjunct to clinical, biochemical, imaging-based, and metabolic markers within integrated diagnostic and prognostic models [45,46,47].

**Table 1:** Selection of studies evaluating nutrigenetic markers, dietary factors, and clinical response in MASLD.

Author, et al., Year	Genetic Marker/Approach	Study Design [N]	Dietary Factor or Pattern Studied	Main Results
Ramos-Lopez, et al., 2020 [37]	Integrative nutrigenetic prototype	Personalized nutrition modeling study in overweight/obese adults	Energy-restricted diets with different macronutrient distribution	An integrative model was proposed to support diet assignment.
Perez-Diaz-Del-Campo, et al., 2021 [39]	*SH2B1* rs7359397	FLiO study; 86 overweight/obese subjects with NAFLD; 6-month dietary intervention	Energy-restricted diet; fiber and omega-3 fatty-acid intake explored by genotype	No-risk genotype showed decreases in hepatic iron and MUFA intake [*p* = 0.047 and *p* = 0.034]. Carriers of the T allele appeared to obtain greater hepatic-health benefit under energy restriction.
Perez-Diaz-Del-Campo, et al., 2021 [38]	Genetic risk scores with 95-variant genetic panel	FLiO study; 86 overweight/obese subjects with NAFLD	Six-month energy-restricted dietary treatment	23 SNPs were associated with change in FLI, 16 with OWLiver-test and 8 with MRI. Regression models explained 53% of FLI improvement, 16% of lipidomic metabolism and 34% of liver fat content; reported significance threshold *p* < 0.05.
Perez-Diaz-Del-Campo, et al., 2022 [40]	95-variant genetic panel	Intervention study; 86 overweight/obese volunteers with NAFLD randomized to AHA or FLiO diets	Energy-restricted AHA or FLiO diet; genetic screening combined with insulin-resistance markers	The FLI-based model personalized the most suitable diet for 72% of volunteers. Diet x genetic score interaction was significant for RBP4 [*p* < 0.005] and TyG index [*p* = 0.009], and remained significant in the MRI liver-fat ≥ 5% subgroup [*p* = 0.001].
Vilar-Gomez, et al., 2021 [48]	*PNPLA3* rs738409	Gene–diet interaction analysis in patients with NAFLD; fibrosis risk assessed in relation to diet and genotype	Carbohydrates, n-3 PUFAs, isoflavones, methionine and choline intake	The *PNPLA3* G allele modulated nutrient–fibrosis associations. Among G-allele carriers, significant fibrosis was associated with carbohydrates [adj. OR 1.04, *p* = 0.019], n-3 PUFAs [adj. OR 0.16, *p* < 0.01], isoflavones [adj. OR 0.65, *p* = 0.025], methionine [adj. OR 0.30, *p* < 0.01] and choline [adj. OR 0.29, *p* < 0.01].
Oh, et al., 2023 [33]	*PNPLA3* rs738409	Korean Genome and Epidemiology Study; observational gene–diet interaction analysis	Sodium intake and baechu kimchi intake; nutrient–food interactions with *PNPLA3*	High sodium intake showed a protective interaction with *PNPLA3* genotype [interaction *p* = 0.002]; baechu kimchi also interacted with PNPLA3 [*p* = 0.012].
Paolini, et al., 2023 [49]	*PNPLA3* I148M/rs738409	Discovery cohort n = 172 with non-invasive NAFLD assessment; validation cohort n = 358 biopsy-proven NAFLD; transcriptomic cohort n = 183	Dietary and circulating niacin; obesity as environmental modifier	Dietary niacin intake and serum niacin were lower in *PNPLA3* CG/GG carriers. In obesity, *PNPLA3* G allele was independently associated with lower serum niacin [discovery beta = −0.21, *p* = 0.04; validation beta = −0.24, *p* = 0.009].
Chen, et al., 2024 [28]	*PNPLA3*, *TM6SF2* and hepatic steatosis PRS	UK Biobank population-based cohort; genotyping, liver fat content and cT1 assessment	Mediterranean diet, fruit/vegetable/legume/fish intake, red/processed meat intake and 24 h dietary recall	Mediterranean diet and higher fruit/vegetable/legume and fish intake were associated with lower liver fat content, while red/processed meat intake and genetic predictors were associated with higher liver fat.
Kwon, et al., 2024 [41]	*GCKR* rs780094 x Mediterranean-diet adherence	Cross-sectional/observational cohort of 33,133 Korean adults	K-MEDAS Mediterranean-diet adherence	Significant interaction between *GCKR* rs780094 and K-MEDAS on MASLD [*p* < 10^−2^]. Among high K-MEDAS participants, minor-allele carriers had lower MASLD risk [OR = 0.88 [0.85–0.91], *p* = 5.54 × 10^−13^].
Liu, et al., 2024 [50]	Polygenic risk score and FIB-4 as modifiers of lifestyle effect	UK Biobank prospective cohort; 417,986 participants free of severe liver disease at baseline; median follow-up 12.6 years	Seven modifiable healthy lifestyle factors	A higher lifestyle score was associated with lower severe liver disease risk [*p* < 0.001]. The protective association was more pronounced among individuals with higher genetic and fibrosis risk; each 1-point lifestyle score combined with 1-SD higher PRS was associated with an additional 2% reduction in SLD risk.
Giardoglou, et al., 2024 [51]	MASLD polygenic risk score optimized for MRI-PDFF prediction	UK Biobank PRS development/validation; 3553 participants with MRI-PDFF; external application in a Greek NAFLD study	Not a dietary intervention; genetic liability integrated with imaging phenotype	An optimal 75-SNP PRS was selected [incremental R^2^ = 0.025, *p* = 0.00145]. The selected PRS predicted LDL cholesterol, diastolic blood pressure and disease outcome in the Greek NAFLD study.
Pafili, et al., 2025 [52]	*PNPLA3* rs738409	Randomized 8-week hypocaloric diet trial in participants with type 2 diabetes; n = 31	Two low-energy diets differing in cereal fiber, coffee and red meat content	*PNPLA3* G-allele carriage modulated diet-induced improvement in liver lipid content, suggesting that genotype may influence response to hypocaloric dietary strategies in type 2 diabetes and steatotic liver disease.
Us, et al., 2025 [42]	*PNPLA3* rs738409	Randomized controlled trial; 12-week MASLD dietary intervention	Mediterranean versus low-fat hypocaloric diets	Both diets were reported as similarly effective for MASLD resolution regardless of *PNPLA3* genotype; exact *p*-values were not available in the accessible abstract/source text.
De Vincentis, et al., 2022 [45]	PRS-HFC based on *PNPLA3*, *TM6SF2*, *MBOAT7* and *GCKR*	UK Biobank prospective cohort; 266,687 individuals; median follow-up 9 years	Genetic enrichment of clinical fibrosis scores	PRS-HFC improved risk stratification and prediction for severe liver disease when added to clinical fibrosis scores, particularly in individuals with metabolic risk factors; AUROC increases were significant for most models.
Kim, et al., 2026 [46]	*PNPLA3* and *TM6SF2* genotypes integrated into non-invasive fibrosis scores	MASLD patients with type 2 diabetes mellitus; advanced fibrosis prediction study	Diagnostic/prediction model in a high-risk metabolic subgroup	Integration of *PNPLA3* and *TM6SF2* genotypes provided incremental improvement in advanced fibrosis prediction among MASLD patients with type 2 diabetes, supporting genetic information as an adjunct to non-invasive diagnostic stratification.

Abbreviations: AHA, American Heart Association; cT1, iron-corrected T1; FIB-4, fibrosis-4 index; FLI, fatty liver index; FLiO, Fatty Liver in Obesity; GCKR, glucokinase regulator; HFC, hepatic fat content; K-MEDAS, Korean Mediterranean-Diet Adherence Screener; MASLD, metabolic dysfunction-associated steatotic liver disease; MRI-PDFF, magnetic resonance imaging-proton density fat fraction; MUFA, monounsaturated fatty acids; NAFLD, non-alcoholic fatty liver disease; OR, odds ratio; PRS, polygenic risk score; RBP4, retinol-binding protein 4; SH2B1, SH2B adaptor protein 1; SLD, severe liver disease; SNP, single-nucleotide polymorphism; TyG, triglyceride-glucose index. NAFLD terminology is retained within individual study descriptions when it corresponds to the terminology used in the original publication.

### 2.2. Nutrigenomics

Nutrigenomics focuses on the mechanisms through which nutrients and dietary patterns modulate gene expression, particularly mRNA abundance, miRNA-mediated post-transcriptional regulation, signaling networks, downstream cellular programs, and functional pathway activity, including KEGG Orthology [KO]-annotated routes involved in lipid metabolism, glucose handling, bile-acid signaling, oxidative stress, inflammation, autophagy, and extracellular matrix remodeling [26,53]. In liver disease, this field is mechanistically central because hepatocytes respond rapidly to carbohydrate flux, dietary fatty-acid composition, fasting–feeding transitions, insulin signaling, bile acid changes, and inflammatory ligands by modifying mRNA expression programs involved in lipogenesis, β-oxidation, oxidative stress, autophagy, inflammation, and fibrogenic remodeling. In parallel, miRNAs provide an additional regulatory layer by repressing or fine-tuning target mRNAs, thereby influencing hepatic lipid metabolism, insulin signaling, inflammatory activation, stellate-cell responses, and extracellular matrix deposition [54]. The main pathways repeatedly highlighted in MASLD include SREBP1c- and ChREBP-driven de novo lipogenesis, pathways regulating mitochondrial and peroxisomal fatty-acid oxidation, endoplasmic reticulum stress responses, innate immune activation, stellate-cell fibrogenesis, and in more advanced disease, senescence-linked and DNA-damage-associated transcriptional states (Table 2). Nutrigenomics is therefore the most attractive framework for moving beyond phenotype labels such as “steatosis” or “MASH” toward biologically interpretable endotypes that may differ in their nutritional responsiveness [30]. This approach is consistent with broader precision-nutrition frameworks proposing that nutrigenomic, epigenomic, metagenomic, proteomic, and metabolomic layers should be integrated to connect dietary exposures with clinically relevant phenotypes and therapy outcomes supported on emerging IA strategies [24]. In MASLD/MAFLD, multi-omics nutritional approaches can help map the biological links between dietary patterns, genetic susceptibility, gut-derived signals, hepatic lipid metabolism, and inflammatory remodeling, thereby supporting a mechanistic rather than purely descriptive interpretation of metabolic liver disease [25]. Experimental dietary preclinical models also support this concept, showing that obesogenic diets can induce hepatic molecular alterations, including increased oxidative stress and diet-induced proteomic changes, before or alongside overt metabolic deterioration involving nutrigenomics mechanisms [55,56].

The difficulty is that the liver, unlike blood, is not easily serially sampled in routine nutritional trials [57]. As a result, many dietary intervention studies in MASLD still rely on body weight, liver enzymes, ultrasound, or MRI-based outcomes rather than repeated liver transcriptional profiling [26]. This situation means that nutrigenomics currently contributes more to disease mapping and biomarker discovery than to prospective assignment of one dietary intervention versus another [57]. Even so, recent translational studies have considerably improved the bridge between hepatic molecular states and clinically scalable biomarkers supported by imaging and IA tools [57].

A notable example is a multicenter study, which integrated paired liver transcriptomics and circulating proteomics in patients with histologically characterized NAFLD and identified a 31-marker proteo-transcriptomic signature associated with active steatohepatitis and advanced fibrosis [57]. The importance of that work is not merely descriptive, suggesting that inaccessible hepatic states may be inferable through circulating surrogates, which is precisely the sort of translational bridge that precision nutrition will need if it is ever to move into routine care [57]. In practical terms, such signatures may help identify whether a patient is dominated by active lipogenesis, inflammatory remodeling, or fibrogenic signaling even when repeated biopsy is neither feasible nor ethical [57]. Within this framework, liver mRNA profiles provide a direct readout of activated metabolic, inflammatory, and fibrogenic programs, whereas miRNAs add a regulatory layer by modulating post-transcriptional gene expression and may also be measured in circulation as candidate non-invasive biomarkers of MASLD activity and response to nutritional intervention [57,58,59]. These findings also link nutrigenomics with nutrimetabolomics. While transcriptomic and proteomic data can indicate which hepatic pathways are activated, metabolomic profiling shows whether these changes are reflected in circulating metabolites, including lipids, amino acids, bile acids, acylcarnitines, or diet-related compounds. Used together, these approaches may help clarify whether a dietary intervention is producing a true biological response, beyond changes in weight, liver enzymes, or self-reported adherence [29,60,61]. That, in turn, opens up the possibility that future nutritional interventions may be layered according to dominant biology, for example, by escalating polyphenol-rich dietary approaches, exercise intensity, or adjunctive pharmacotherapy in patients with persistent inflammatory or fibrogenic hepatic signatures despite weight loss [62].

A second major contribution comes from model benchmarking. In this sense, a study compared murine dietary MASLD models with human molecular disease and showed that not all diet-induced models reproduce the human transcriptional architecture with equal fidelity [62]. If a dietary model generates steatosis without reproducing the inflammatory, fibrogenic, or metabolic pathways seen in human disease, its apparent “diet response” may be mechanistically misleading [62].

Human computational integration studies reinforce this point since a large integrative analysis of ten transcriptomic datasets, including 903 liver tissue samples across different NAFLD stages, identified epigenetic and epitranscriptomic programs associated with disease progression and fibrosis [30,63]. Consistently, hepatic mRNA signatures linked to inflammatory injury and fibrogenesis include genes involved in macrophage recruitment/activation and cytokine signaling, such as CCL2, CXCL10, TNF, IL1B, and CD68, as well as extracellular-matrix remodeling and scar deposition genes, including COL1A1, COL3A1, TIMP1, MMP2, TGFB1, and THBS2 [64,65]. In parallel, steatosis and hepatic lipid handling have been associated with altered expression of lipid uptake, storage, and lipogenesis-related transcripts such as CD36, SCD, FASN, DGAT2, PNPLA3, and PLIN2 [65]. Such analyses suggest that patients may enter superficially similar clinical states through different transcriptional routes, which is one of the strongest arguments for molecular stratification in future diet trials [30]. In other words, nutrigenomics may not yet provide a sufficiently robust basis for prescribing a genotype-specific diet in routine clinical practice, but it is increasingly informative for understanding why two patients with similar MRI-PDFF or ALT values may require different levels of follow-up intensity and adjunctive management [30,57]. This interpretation also links nutrigenomics with metabolomics and functional liver testing. While transcriptomic analyses identify altered hepatic programing affecting lipid metabolism, inflammation, and cell functions, metabolomic approaches can capture their downstream biochemical expression through circulating lipids, amino acids, bile acids, and other diet-related metabolites. Serum lipidomic platforms such as OWLiver may help translate these molecular differences into non-invasive diagnostic and monitoring tools for MASLD-related phenotypes, including MASH and fibrosis, whereas liver breath tests could provide complementary information on hepatocellular metabolic function [66]. Together, these approaches may bring molecular stratification closer to clinically usable diagnosis and follow-up, without implying that transcriptomics alone is ready for routine diet prescription [66,67,68,69].

**Table 2:** Selection of studies evaluating nutrigenomic, transcriptomic, and proteomic responses to dietary or disease-related liver remodeling in MASLD/MASH.

Author, et al., Year	Omics Platform/Tissue	Study Design [N]	Dietary Factor or Pattern Studied	Main Results
Milagro, et al., 2006 [55]	Gene-expression and oxidative-stress assessment; rat liver and white adipose tissue.	Experimental diet-induced obesity study in rats	High-fat feeding model.	High-fat feeding was reported to increase liver oxidative stress; significance reported in the article/figures with *p* < 0.05.
Bondia-Pons, et al., 2011 [56]	Liver proteomic analysis; Wistar rat liver proteome.	Experimental feeding study in Wistar rats	5-week starch-rich control, high-fat, high-sucrose and high-fat-sucrose diets.	17 proteins were altered by >1.25-fold; Hspa8 and Hspa9 were significantly downregulated after high-fat-sucrose diet vs. control [*p* < 0.05].
Suppli, et al., 2019 [68]	Hepatic mRNA transcriptomics/RNA-seq; human liver biopsies.	Cross-sectional transcriptomic study; 57 subjects: healthy normal-weight [n = 14], obese [n = 12], NAFL [n = 15] and NASH [n = 16].	Disease-stage comparisons across healthy normal-weight, obese, NAFL and NASH groups rather than direct dietary intervention.	NAFL/NASH liver samples differed from controls, with 8244 DEGs and perturbations in lipid metabolism, immunomodulation, extracellular-matrix remodeling and cell-cycle control.
Govaere, et al., 2023 [57]	Paired liver transcriptomics and circulating proteomics; liver tissue with matched plasma.	Multicenter biomarker/transcriptomic study; 306 patients with histologically characterized NAFLD.	Not a dietary intervention; biopsy-characterized NAFLD used for biomarker discovery.	4730 circulating proteins were profiled and integrated with paired liver transcriptomics; a 31-marker proteo-transcriptomic signature was developed.
Herranz, et al., 2023 [30]	Integrated human liver transcriptomic meta-analysis; 903 liver samples.	Integrated transcriptomic meta-analysis; 10 datasets and 903 liver tissue samples.	Disease-stage comparisons across normal liver, obesity, NAFL and NASH rather than direct dietary intervention.	Ten transcriptomic datasets were integrated to identify epigenetic and epitranscriptomic gene-expression programs linked to progression.
Vacca, et al., 2024 [62]	Cross-species RNA-seq benchmark of dietary mouse models.	Cross-species RNA-seq benchmarking study of dietary MASLD/MASH mouse models versus human disease	High-fat, high-fructose, cholesterol-enriched, choline-deficient and other MASLD/MASH dietary models.	Western diets aligned more closely with human MASH; high cholesterol, longer study duration and/or genetic manipulation intensified liver damage and F2+ fibrosis.
Tobaruela-Resola, et al., 2024 [58]	Circulating miRNA profiling with inflammatory, depressive, hepatic and metabolic variables; plasma/serum.	FLiO longitudinal study; 55 subjects with MASLD, assessed at baseline and after 6, 12 and 24 months.	Two-year weight-loss-oriented nutritional intervention; longitudinal hepatic, metabolic, inflammatory and psychological assessment.	Panels combining miRNAs and clinical variables predicted MASLD after intervention; 24-month panels reached AUC 0.90 and 0.89, including liver stiffness or hepatic fat, lipids, BMI/depressive symptoms and miR15b-3p.
Tobaruela-Resola, et al., 2025 [59]	Circulating miRNA profiling by RT-PCR; plasma/serum samples.	Longitudinal biomarker study in subjects with MASLD after a 2-year nutritional intervention.	Six-, 12- and 24-month dietary follow-up; miRNA panels evaluated with BMI and MASLD resolution status.	Intervention modulated miRNAs over time. Panels for MASLD disappearance reached AUC 0.68 at 6 months [miR15b-3p/miR126-5p/BMI], AUC 0.85 at 12 months [miR29b-3p/miR122-5p/miR151a-3p/BMI] and AUC 0.85 at 24 months [miR21-5p/miR151a-3p/BMI].

Abbreviations: F2+, fibrosis stage 2 or higher; Hspa, heat shock protein; MASLD, metabolic dysfunction-associated steatotic liver disease; MASH, metabolic dysfunction-associated steatohepatitis; N, sample size; NAFL, non-alcoholic fatty liver; NAFLD, non-alcoholic fatty liver disease; NASH, non-alcoholic steatohepatitis; RNA-seq, RNA sequencing.; DEG, differentially expressed gene; mRNA, messenger RNA; miRNA, microRNA; RT-PCR, reverse transcription polymerase chain reaction. NAFLD terminology is retained within individual study descriptions when it corresponds to the terminology used in the original publication.

### 2.3. Nutriepigenetics

Nutriepigenetics investigates the interaction of dietary exposures influencing gene regulation without altering the underlying DNA sequence, typically through DNA methylation, histone modification, chromatin remodeling, non-coding RNAs, and epitranscriptomic mechanisms [69,70,71]. In liver disease, this field is especially appealing because it provides a plausible memory layer linking long-term nutritional exposure to durable differences in steatosis, inflammation, and fibrogenesis [72]. Diet can influence this layer through one-carbon metabolism and methyl-donor availability, oxidative stress, acetyl-CoA flux, NAD+-dependent deacetylation pathways, lipid peroxidation, and small-RNA signaling [73]. In MAFLD/MASLD, methylation-related nutri-epigenetic mechanisms have been specifically proposed as regulatory routes through which dietary factors may influence hepatic lipid accumulation, inflammation, insulin resistance, and disease progression [74]. In principle, nutriepigenetics is attractive because it may capture not just what a patient eats today, but the biological residue of what they have been exposed to over months or years [72].

The strongest recent human evidence has come from blood-based methylation signatures and circulating miRNA studies [75]. Diet-related methylation changes in blood have been shown to stratify liver biopsy fibrosis grades in MASLD [72,76]. That observation is translationally important because it suggests that nutritional exposures leave systemic epigenetic marks with relevance to hepatic disease severity [72]. Even if peripheral blood does not perfectly mirror the hepatic epigenome, a robust blood signature could still be clinically useful as it improves non-invasive risk discrimination [72]. This approach is crucial distinction, because clinical utility does not require full biological identity between tissues; it requires reproducible and clinically meaningful correlation [70].

At the same time, integrative molecular work has shown that epigenetic dysregulation is embedded in disease progression rather than representing peripheral noise. In MASLD and alcohol-related liver disease, differential DNA methylation at specific CpG sites within fibrosis-related genes has been reported to distinguish patients with mild versus advanced fibrosis, supporting the potential value of methylation sites as part of broader molecular panels for fibrosis-risk stratification [77]. Nutriepigenetics therefore occupies a distinctive conceptual niche bringing sustained nutritional exposure with relatively durable biological remodeling, placing it between genetic predisposition and metabolic readouts [27].

Longitudinal evidence is also beginning to emerge concerning nutriepigenetic interaction and clinical application in liver diseases [78]. Evidence from longitudinal nutritional interventions suggests that circulating miRNA signatures may contribute to non-invasive MASLD assessment. In this context, panels combining circulating miRNAs with inflammatory and behavioral variables improved MASLD discrimination during a two-year nutritional intervention, while related work proposed post-intervention circulating miRNA panels as potential minimally invasive markers for MASLD monitoring [58,59]. These findings are particularly relevant because they point to a role for nutriepigenetics not primarily in baseline diet assignment, but in longitudinal response monitoring, where the clinical question is whether a patient is biologically improving despite imperfect changes in weight or liver enzymes [58,59]. This information could be valuable in patients with discordant outcomes, such as apparent weight-loss success but persistent hepatic inflammation, or conversely modest weight change with unexpectedly favorable hepatic remodeling [79].

The limitations, however, are substantial and should be stated explicitly about nutrigenetics clinical applications. Cell-type specificity remains a major challenge, because DNA methylation patterns measured in peripheral blood cells or circulating miRNA concentrations may reflect immune activation, adipose tissue remodeling, or medication effects rather than liver-specific biology [72]. For example, diet-related methylation changes detected in blood cells have been reported to stratify liver biopsies from NAFLD patients according to fibrosis grade, but such peripheral signatures still require careful interpretation because they may capture systemic inflammatory or metabolic remodeling rather than liver-specific epigenetic regulation alone [70]. Similarly, circulating miRNA panels measured after a two-year nutritional intervention, including miR-122-5p, miR-151a-3p, miR-126-5p, miR-21-5p, and miR-15b-3p, have been proposed as non-invasive MASLD biomarkers, but these panels included clinical, inflammatory, behavioral, and metabolic variables in addition to miRNAs, emphasizing that epigenetic readouts are most informative when integrated with broader phenotyping rather than used in isolation [58,59]. Reverse causation is also difficult to exclude, since severe liver disease may itself alter epigenetic marks [30]. Assay normalization and cross-platform reproducibility remain imperfect [58]. Moreover, while methyl-donor biology and histone acetylation offer compelling mechanistic narratives, the evidence base is not yet sufficient to justify disease-stage-specific prescription of methyl-donor-rich or epigenetically targeted diets on the basis of peripheral assays alone [80]. As an experimental example, methyl-donor supplementation with nutrients such as betaine, choline, vitamin B12, and folic acid reduced hepatic triglyceride accumulation and modified the DNA methylation profile of fatty acid synthase in a preclinical model involving a obesogenic diet [71]. Related experimental findings also showed that methyl-donor supplementation can modify hepatic transcriptomic and epigenetic responses to obesogenic diets and reduce liver fat accumulation, supporting the biological plausibility of diet-sensitive epigenetic programming in fatty liver disease [76]. Related perinatal studies also suggest that maternal methyl-donor availability during lactation can influence offspring metabolic and hepatic features, reinforcing the concept that nutritional exposures may leave durable regulatory effects beyond the immediate dietary period [77]. Thus, the most plausible current role of nutriepigenetics is as a minimally invasive longitudinal adjunct that may help detect non-response earlier and refine monitoring intensity, rather than as a mature instrument for front-end diet selection [59].

### 2.4. Nutrimetagenomics

Nutrimetagenomics examines the role of diet in shaping the composition and function of the gut microbiome and how that microbiome, in turn, may modify host responses to diet [81,82,83]. In hepatology this field has immediate biological associations because the portal circulation directly links the gut and the liver, exposing hepatocytes and non-parenchymal cells to short-chain fatty acids, secondary bile acids, choline metabolites, endotoxin, ethanol-like products, and inflammatory signals derived from altered intestinal permeability [84]. The attraction of this field is that it captures an important reason to explain because two patients consuming apparently similar diets may generate different metabolic and inflammatory consequences at the hepatic level [83,85]. This interpretation is consistent with recent precision-medicine frameworks in metabolic liver disease, which emphasize that gut microbial functions, host genetics, and dietary exposures interact to shape metabolic phenotypes and may help identify patient subgroups with distinct preventive or therapeutic needs [86]. Therefore, such an outcome is one of the most actively translational omics domains in MASLD [87].

The randomized DIRECT-PLUS trial represents a key example explaining that interventions can combine clinically meaningful imaging endpoints with mechanistic exploration [83]. In adults with abdominal obesity or dyslipidemia, a green-Mediterranean dietary pattern achieved greater reductions in MRI-measured intrahepatic fat than comparator dietary strategies [83]. Beyond demonstrating an effect on liver fat, the trial also provided a framework for subsequent mechanistic analyses linking dietary composition, adiposity changes, and molecular responses [83]. Within the same trial framework, cardiometabolic improvements were subsequently associated with gut microbiome remodeling, supporting the possibility that part of the hepatic and metabolic benefit of dietary intervention may be mediated through functional changes in the microbiome [83].

Observational and intervention-adjacent datasets support the same general direction [88,89]. Similarly, a PREDIMED-Plus subanalysis showed that lifestyle-induced changes in gut microbiota composition, including diet-sensitive microbial taxa and community patterns, paralleled improvements in non-invasive steatosis and fibrosis indices, supporting the relevance of microbiota remodeling as a biological correlate of hepatic response [82,90]. In patients with MASLD and type 2 diabetes, gut barrier dysfunction and host–microbiome interactions have also been associated with greater disease severity [31]. Together, these studies argue that generic advice to “eat more fiber” is biologically incomplete, because the clinically relevant question is not only which foods enter the gut, but how the gut microbiota transforms dietary substrates into microbial functions and metabolites that influence hepatic pathways [84,86].

Interventional microbiome-modifying strategies have yielded mixed results, and that inconsistency is itself instructive. In a randomized trial in NASH, probiotic supplementation altered microbiota profiles and improved fibrosis-related parameters [86]. By contrast, a randomized pilot trial evaluating prebiotic treatment in patients with NAFLD illustrated the challenges of obtaining clinically meaningful liver benefits from microbiome-targeted interventions alone [87]. Thus, the microbiome may act as an important mediator of dietary response, but in many patients, it is unlikely to represent an independent therapeutic target unless excess energy intake, adipose tissue insulin resistance, and broader cardiometabolic dysfunction are addressed in parallel [86,87].

Several factors still limit the direct clinical translation of nutrimetagenomic findings. Although MASLD studies have reported disease-related shifts in taxa such as Veillonellaceae, Ruminococcaceae, Enterobacteriaceae, Ruminococcus, Faecalibacterium, Coprococcus, Lachnospira, Citrobacter, Megamonas, Bacteroides, Escherichia, and Klebsiella, as well as functional pathways involving bile-acid metabolism, ethanol fermentation, lipopolysaccharide/peptidoglycan biosynthesis, branched-chain amino-acid metabolism, and short-chain fatty-acid production, these signals remain highly context-dependent [88]. Nevertheless, specific gut microbiome and metabolite signatures have been associated with significant fibrosis in non-obese NAFLD, suggesting that microbiome-derived information may contribute to disease stratification [89]. In addition, stool-based profiles may not fully capture mucosa-associated communities or proximal small-intestinal processes that could be more directly involved in host–microbiome crosstalk [90]. Because many reported associations remain insufficient to establish directionality, current evidence supports nutrimetagenomics primarily as a tool for mechanistic enrichment and response monitoring rather than as a standalone basis for clinical decision-making [88,89,90]. Its robust implementation in MASLD will likely require longitudinal sampling, functional characterization, and integration with other omics and clinical layers, rather than reliance on single-time-point stool taxa alone [84].

### 2.5. Nutrimetabolomics

Nutrimetabolomics profiles small molecules produced by the interaction the genetic make-up of diet, host metabolism, microbiome activity, and disease state [29]. Among all nutritional omics domains, this may be the closest to routine clinical use because plasma, serum, urine, and sometimes stool are comparatively easy to sample repeatedly [61]. Metabolites are also attractive because they capture both exposure and response, thereby solving a central problem in lifestyle medicine, namely the inaccuracy of self-reported adherence [29]. From a broader precision-medicine perspective, nutrimetabolomics has also been proposed as a practical strategy to characterize individual metabolic profiles, evaluate the interaction between diet, organismal function, and environmental exposures, and support more personalized nutritional and health interventions [91]. In liver disease, metabolomics can reveal lipotoxicity, mitochondrial stress, ketone and acylcarnitine flux, amino-acid dysregulation, inflammatory lipid remodeling, and bile-acid perturbation even when standard liver enzymes are relatively uninformative [60].

Recent evidence indicates that metabolomic signatures can act as objective bridges between diet patterns and hepatic phenotypes. An 81-metabolite signature reflecting adherence to the EAT-Lancet dietary pattern, including metabolites related to fatty-acid composition and unsaturation, omega-3 fatty acids such as docosahexaenoic acid, saturated fatty-acid percentage, glycoprotein acetyls, albumin, and lipoprotein-related traits, has been associated with a lower risk of MASLD [29]. This is important because it turns dietary pattern analysis from a purely questionnaire-based construct into a biologically anchored exposure metric [29]. Moreover, Mediterranean diet-associated metabolite signatures, particularly small HDL particles and omega-3 fatty acids, have further been linked to MASLD progression and liver-related outcomes in prospective analyses [61].

Nutrimetabolomics also provides insight into disease staging and underlying pathophysiology. In adults with severe obesity, distinct metabolic signatures have been associated with MASLD severity, while plasma lipidomic profiles appear to vary across MASLD stages, with specific phospholipid species emerging as potential stage-sensitive biomarkers [92]. A critical interpretive point is that metabolomic responses are not driven by diet alone. Exercise intervention in NAFLD has been shown to induce metabolic changes across adipose tissue, plasma, urine, and stool [93]. These observations matter clinically because patients who achieve similar weight loss may still have very different residual states of mitochondrial inefficiency, membrane remodeling, or inflammatory lipid flux [60]. Metabolomics therefore offers a way to distinguish superficial lifestyle success from true biological remission [79]. In this context, OWL-type serum lipidomic tests represent one of the most clinically oriented applications of metabolomics, as they use fasting blood lipid profiles, together with clinical variables, to support non-invasive discrimination between normal liver, simple steatosis, steatohepatitis, and fibrosis-related phenotypes [66].

This complexity also illustrates the broader role of metabolomics in precision nutrition. Metabolomic profiling has been proposed as a means of improving dietary-exposure assessment, characterizing inter-individual response, and supporting the delivery of more precise nutritional strategies [94]. In practical terms, the most useful clinical application may therefore be repeated assessment of whether a patient’s biological trajectory is moving in the expected direction during a combined diet-and-lifestyle intervention. Serum lipidomic approaches and other metabolite-based tools may additionally contribute to non-invasive disease characterization when interpreted together with conventional clinical variables [60,66].

At present, the main barriers to translation include intervention-study design, analytical heterogeneity, fasting status, preanalytical handling, batch effects, and reproducibility across populations and platforms. Few studies have yet tested whether prospective metabolomic phenotyping can guide dietary selection and improve liver-related outcomes beyond standard clinical care. Accordingly, the near-term value of nutrimetabolomics is likely to lie in objectively characterizing metabolic status, dietary exposure, and biological response, identifying potential non-response, and supporting adaptive follow-up rather than independently determining a specific dietary prescription [29,61,94].

### 2.6. Translating and Integrating Omics into Clinical Practice

Taken together, these five domains are best understood as complementary layers within a precision-nutrition approach to hepatology, rather than as separate or competing paradigms [27], which are expected to benefit from IA supervision. Each omics domain contributes a distinct type of information: nutrigenetics defines inherited susceptibility; nutrigenomics characterizes dominant hepatic transcriptional programs; nutriepigenetics reflects durable regulatory remodeling and exposure memory; nutrimetagenomics captures host–microbiome processing of diet; and nutrimetabolomics offers a dynamic, repeatable readout of biological response and suitable metabolomic assessments. Precision nutrition should therefore remain phenotype-first, using omics selectively to clarify why a given phenotype has emerged and whether it is changing under treatment [28,57,62,72,83]. Within this translational pathway, a 4P precision-nutrition model could use nutriomics to support predictive risk estimation, preventive lifestyle targeting, personalized endotype-based monitoring, and participative long-term management. In practical terms, genetic and clinical risk profiles may inform prediction, early metabolic and microbiome alterations may guide prevention, transcriptomic, epigenetic, metagenomic, and metabolomic patterns may refine personalization, and repeated biomarker feedback may strengthen patient participation and adherence over time.

This perspective also underscores the need to avoid overstating the current degree of personalization that nutritional omics can support. At present, the evidence most strongly supports omics-guided risk enrichment and monitoring, not fully individualized prescription of specific foods on the basis of molecular testing alone [42,79]. A clinically realistic pipeline would therefore use inexpensive phenotyping and standard fibrosis assessment first, then reserve deeper omics for patients with discordant risk, severe cardiometabolic burden, early-onset disease, unexpected non-response, or uncertainty regarding the dominant mechanism [28,57]. This stepwise approach is particularly relevant for implementation in hepatology services, where resources, biopsy access, and reimbursable remain limiting factors [14,15]. Indeed, nutriomic tools provide promising outcomes concerning early diagnosis, founded prognosis, patient stratification and precision management.

Future studies should move beyond retrospective biomarker discovery and test whether omics-informed strategies improve clinical outcomes compared with standard care. This will require multiethnic recruitment, standardized dietary assessment, repeated biospecimen collection, prespecified omics-stratified hypotheses, and clinically meaningful endpoints such as MRI-PDFF, cT1, fibrosis biomarkers, histology when appropriate, and liver-related outcomes. Importantly, omics should also be evaluated as tools for adaptive care, supporting escalation or de-escalation of lifestyle, pharmacological, or monitoring intensity according to biological response over time. Artificial intelligence may be useful in this context by integrating high-dimensional omics, imaging, clinical, dietary, and microbiome data into interpretable prediction models as well as imaging echography or FibroScan technologies as integrated with AI. In liver disease, AI-based analysis of omics data has been proposed as a route toward more personalized diagnosis, prognosis, and treatment, particularly when molecular layers are integrated with phenotypic and clinical information [95]. However, its clinical value will depend on external validation, transparency, bias assessment, and demonstration that AI-guided decisions improve outcomes beyond conventional risk scores [96,97]. The potential diagnostic value of integrating molecular information is also illustrated by the development of a gene signature for the diagnosis of MASLD in metabolically unhealthy individuals with obesity [98].

Finally, translation beyond MASLD into alcohol-related liver disease, viral hepatitis, cholestatic disorders, and compensated cirrhosis will require disease-specific validation rather than extrapolation, because the metabolic context, inflammatory architecture, and host–microbiome environment differ substantially across liver diseases.

## 3. Conclusions

Nutritional omics has moved precision nutrition in hepatology from a conceptual aspiration toward a biologically grounded translational field. The strongest evidence currently comes from MASLD/MASH, where genetic susceptibility, hepatic transcriptional programs, epigenetic remodeling, gut microbial function, tissue biopsies and circulating metabolites reveal clinically meaningful heterogeneity involving imaging instrumentation. However, current evidence does not yet support fully individualized diet prescription based on molecular testing alone. Rather, the most suitable near-term role of nutritional omics is to improve diagnosis and risk stratification, biological interpretation, and longitudinal monitoring of treatment response. In this context, artificial intelligence and machine learning approaches may facilitate the integration of high-dimensional omics, imaging, clinical, dietary, and microbiome data, identify complex multivariable patterns, and support biomarker discovery and molecular endotyping. These approaches may therefore help transform heterogeneous biological information into clinically interpretable models for precision nutrition. However, their clinical value will depend on external validation, reproducibility, transparency, bias assessment, and demonstration of added value beyond conventional clinical predictors.

Framed within 4P precision nutrition, nutriomic may help move hepatology toward predictive, preventive, personalized, and participative models of chronic liver disease care. In this context, integrated nutriomic approaches could support prediction of disease risk and progression, prevention through earlier lifestyle targeting, personalization through endotype- and response-based monitoring, and participation through repeated biomarker and imaging feedback that strengthens patient engagement and long-term adherence. Precision nutrition in hepatology is therefore unlikely to emerge from any single omics platform, but from integrated, phenotype-first models that combine realistic lifestyle interventions with stable susceptibility markers, mechanistic endotyping, and repeated biological monitoring, where advancing in artificial intelligence will pave the way for preventive, prescription and personalization nutritional and clinical interventions involving precision clinical hepatology.

## Data Availability

No new data were created or analyzed in this study. Data sharing is not applicable to this article.

## Abbreviations

The following abbreviations are used in this manuscript:

4P	Predictive, preventive, personalized, and participatory
AHA	American Heart Association
AI	Artificial intelligence
ALT	Alanine aminotransferase
AUC	Area under the curve
AUROC	Area under the receiver operating characteristic curve
BMI	Body mass index
CCL2	C-C motif chemokine ligand 2
CD36	Cluster of differentiation 36
CD68	Cluster of differentiation 68
ChREBP	Carbohydrate-responsive element-binding protein
CoA	Coenzyme A
COL1A1	Collagen type I alpha 1 chain
COL3A1	Collagen type III alpha 1 chain
CpG	Cytosine–phosphate–guanine dinucleotide
cT1	Iron-corrected T1
CXCL10	C-X-C motif chemokine ligand 10
DALY	Disability-adjusted life year
DEG	Differentially expressed gene
DGAT2	Diacylglycerol O-acyltransferase 2
DIRECT-PLUS	Dietary Intervention Randomized Controlled Trial PoLyphenols Unprocessed
DNA	Deoxyribonucleic acid
F2+	Fibrosis stage 2 or higher
FASN	Fatty acid synthase
FIB-4	Fibrosis-4 index
FLI	Fatty liver index
FLiO	Fatty Liver in Obesity
GBD	Global Burden of Disease
GCKR	Glucokinase regulator
HDL	High-density lipoprotein
HFC	Hepatic fat content
HSD17B13	Hydroxysteroid 17-beta dehydrogenase 13
Hspa8	Heat shock protein family A member 8
Hspa9	Heat shock protein family A member 9
IL1B	Interleukin 1 beta
KEGG	Kyoto Encyclopedia of Genes and Genomes
K-MEDAS	Korean Mediterranean-Diet Adherence Screener
KO	KEGG Orthology
LDL	Low-density lipoprotein
MAFLD	Metabolic dysfunction-associated fatty liver disease
MASH	Metabolic dysfunction-associated steatohepatitis
MASLD	Metabolic dysfunction-associated steatotic liver disease
MBOAT7	Membrane-bound O-acyltransferase domain-containing 7
miRNA	MicroRNA
MMP2	Matrix metalloproteinase 2
MRI	Magnetic resonance imaging
MRI-PDFF	Magnetic resonance imaging-proton density fat fraction
mRNA	Messenger RNA
MUFA	Monounsaturated fatty acid
N	Sample size
NAD+	Nicotinamide adenine dinucleotide, oxidized form
NAFL	Non-alcoholic fatty liver
NAFLD	Non-alcoholic fatty liver disease
NASH	Non-alcoholic steatohepatitis
OR	Odds ratio
PLIN2	Perilipin 2
PNPLA3	Patatin-like phospholipase domain-containing protein 3
PREDIMED-Plus	PREvención con DIeta MEDiterránea-Plus
PRS	Polygenic risk score
PRS-HFC	Polygenic risk score for hepatic fat content
PUFA	Polyunsaturated fatty acid
RBP4	Retinol-binding protein 4
RNA	Ribonucleic acid
RNA-seq	RNA sequencing
RT-PCR	Reverse transcription polymerase chain reaction
SCD	Stearoyl-CoA desaturase
SD	Standard deviation
SH2B1	SH2B adaptor protein 1
SLD	Severe liver disease
SNP	Single-nucleotide polymorphism
SREBP1c	Sterol regulatory element-binding protein 1c
TGFB1	Transforming growth factor beta 1
THBS2	Thrombospondin 2
TIMP1	Tissue inhibitor of metalloproteinases 1
TM6SF2	Transmembrane 6 superfamily member 2
TNF	Tumor necrosis factor
TyG	Triglyceride-glucose index
VLDL	Very-low-density lipoprotein
